# Biological Markers and Response to Neoadjuvant Taxane-Based Chemotherapy in Patients with Locally Advanced Breast Cancer

**DOI:** 10.5402/2012/245891

**Published:** 2012-12-17

**Authors:** Mohamed I. El-sayed, Doaa W. Maximous, Madeha M. Zakhary, Nabiel N. H. Mikhail

**Affiliations:** ^1^Department of Radiation Oncology, South Egypt Cancer Institute, Assiut University, Assiut, Egypt; ^2^Department of Surgical Oncology, South Egypt Cancer Institute, Assiut University, Assiut, Egypt; ^3^Department of Biochemistry, Faculty of Medicine, Assiut University, Assiut, Egypt; ^4^Department of Biostatistics and Cancer Epidemiology, South Egypt Cancer Institute, Assiut University, Assiut, Egypt

## Abstract

*Introduction*. Biological markers as Her2/neu, p53, and hormonal receptors (HmRs) may be reliable parameters for prognostic assessment of patients of locally advanced breast cancer (LABC). This work aims at assessing the potential value of these biological markers for the prediction of disease outcome after neoadjuvant taxane-based chemotherapy and its implication on the surgical role. *Patients and Methods*. From March 2006 to September 2011, 95 patients with LABC were treated by neoadjuvant taxane-based chemotherapy given at intervals of 3 weeks. Expression of Her2/neu and p53 was examined in the initial tissue biopsy by using ELISA technique. Status of HmRs was determined using a commercial enzyme immunoassay. Three weeks after the third cycle, patients underwent surgical resection followed by 3 more cycles of taxane-based chemotherapy and radiotherapy as an adjuvant therapy. Relations of Her2/neu overexpression to p53, HmRs, and conventional prognostic factors were analyzed. *Results*. Median followup was 61 months. The 5-year DFS and OAS rates were significantly higher in patients with positive HmRs than in those with negative HmRs, patients with Her2− than those with Her2+ breast cancer, and patients with intact p53 breast cancer than those with inactive p53. HER-2 overexpression was statistically significant associated with loss of HmR positive immunostaining (*P* < 0.0001), grade III breast cancer (*P* < 0.0001), advanced nodal status (*P* = 0.0039), and younger (<50 years) age (*P* = 0.0108). *Conclusion*. Her2/neu overexpression was associated with poor DFS and OAS rates, as it was significantly associated with negative HmR and high grade.

## 1. Introduction

Neoadjuvant chemotherapy (NAC) is the standard of care in patients with locally advanced breast cancer (LABC) [1], as it improves local control and survival [2, 3]. The reported clinical response rate to NAC varied between 30% and 90% and the 5-year overall survival (OAS) was reported to range between 40% and 60% [4]. As the clinical and pathological responses of breast cancer to NAC are short-term markers for a long outcome, it is important to identify biological factors that may predict response to NAC and subsequent disease-free survival (DFS) and OAS [5, 6]. It is well established that the expression of the estrogen receptor (ER) and progesterone receptor (PR) determines the responsiveness of tumors to hormonal interventions. Nevertheless the absence or presence of these hormonal receptors does not predict response to chemotherapy [7]. However, breast cancer patients with tumors that are ER positive and/or PR positive have lower risks of mortality after their diagnosis compared to women with ER- and/or PR-negative disease [8, 9]. One of the most important markers being studied is Her2/neu protooncogene, which has been localized to long arm of chromosome and encodes a transmembrane tyrosine kinase growth factor receptor. The protein product of HER-2/neu is overexpressed in 25%–30% of breast cancers [10] and is associated with a poor prognosis [11]. The tumor suppressor gene, p53, is located on short arm of chromosome 17. Mutations in the tumor suppressor gene p53 are present in 18%–25% of primary breast carcinomas [12]. Preclinical data have shown that cells with mutated p53 might be resistant to radiation or chemotherapeutics [13].

The aim of this study is to evaluate the potential value of biological markers (Her2/neu, p53, and hormonal receptor status) for the prediction of disease outcome and response to therapy in patients with locally advanced breast cancer, after neoadjuvant chemotherapy. 

## 2. Patients and Methods

This prospective phase II study was conducted at South Egypt Cancer Institute, Assiut University, Egypt, and Sohag Cancer Centre, Sohag University, Egypt, during the period from March 2006 to September 2011. Informed consent was obtained for all patients, and the protocol was approved by institutional review board at our center.

### 2.1. Patients and Tumor Characteristics

 This study included ninety-five female patients with biopsy proven locally advanced, nonmetastatic breast cancer (stage IIB (limited to T3N0) and stage IIIA disease), Eastern Cooperative Oncology Group performance score of 0 to 1, and left ventricular ejection fraction (LVEF) of ≥60%. Each patient was subjected to clinical examination, laboratory investigations, and breast sonomammography to exclude multiple scattered microcalcifications. True-cut needle biopsy was taken for histopathological diagnosis and assessment of biological factors. Radiological studies were done, such as chest X-ray and abdominopelvic ultrasonography to exclude patients with visceral metastasis and bone scan to exclude patients with bone metastasis. Each patient was given 3 cycles of taxenes based combination chemotherapy with 3 weeks interval, Paclitaxel 135 mg/m^2^, Adriamycin 50 mg/m^2^ and Cyclophosphamide 500 mg/m^2^. Three weeks after the third cycle, each patient was evaluated by clinical examination and breast sonomammography to assess the response to neoadjuvant chemotherapy. Primary end points were evaluation of the value of biological markers for the prediction of response to therapy and survival. Secondary end points were determination of the association of HER-2/neu overexpression in relation to p53 inactivation, hormonal receptor status, and conventional prognostic factors.

### 2.2. Evaluation of Biological Markers

After true-cut needle biopsy, the tissue samples were pulverized while frozen in a chilled mortar and homogenized for 2 minutes in TED buffer in melting ice using a Teflon homogenizer. These homogenates were centrifuged for 10 minutes at 10,000 xg at 4°C. The supernatants were recentrifuged at 3000 xg for another 15 minutes and finally stored at −70°C in 200 mi aliquots till assay. Tissue levels of Her2/neu were determined by ELISA, using the human neu oncoprotein ELISA kit supplied by Oncogene Science Inc., OIA-04, Uniondale, New York, USA. For tissue homogenates, 100 *μ*L of tissue homogenate was extracted with 20 *μ*L of antigen extraction agent supplied with the kit. The tubes were centrifuged at 1500 xg for 10 minutes. The supernatants were diluted 50 times with sample dilution buffer supplied in the kit. Then, 100 *μ*L of diluted sample was used for the determination of Her2/neu. Tissue levels of p53 were also determined by ELISA technique, using kits supplied by Diaclone Research, France, Catalogue number 043. A monoclonal antibody specific for p53 had been coated onto the wells of the microtiter strips provided. During the first incubation, the p53 antigen was added to the wells. After washing, a biotinylated monoclonal antibody specific for p53 was incubated. Then the streptavidin-peroxidase enzyme was added. After incubation and washing to remove all unbound enzymes, a substrate solution which acts on the bound enzyme was added to induce a colored reaction product. The intensity of this colored product is directly proportional to the concentration of p53 present in the samples. Protein concentration in the tissue homogenates was determined by the method of Lowry et al. [14] as modified by Miller [15], whose method combines the Biuret and Folin Ciocalteu reactions [16]. The receptor status had been determined using a commercial enzyme immunoassay according to the instructions of the manufacturer (Abbott Laboratories, Chicago, IL, USA). A result exceeding 15 fmol/mg was considered positive for the presence of the particular receptor.

### 2.3. Assessment of Response

 After neoadjuvant chemotherapy, patients with tumors <5 cm in greatest dimension (objective response) underwent conservative breast surgery (excision of tumor with 2 cm rim of normal breast tissue and level I and II axillary dissection), whereas those with breast tumors ≥5 cm in greatest dimension (no response) or those with persistent positive surgical margins (after CBS) were subjected to MRM. Each patient in both groups was then given additional 3 cycles of taxanes-based combination chemotherapy followed by consolidative radiotherapy of 50 Gys with 2 Gys daily fraction to breast and chest wall using 3D radiation therapy planning (in patients who underwent CBS) and to chest wall using 2D radiation therapy planning (in patients who underwent MRM) by 2 parallel opposed tangential fields using 6 MV photon beams. Supraclavicular irradiation (50 Gys/25 fractions/5 weeks) was given only to patients with positive axillary lymph nodes. A boost dose of 16 Gys in 8 fractions to tumor site using 12 Mev electrons was given to patients who underwent conservative surgery.

All patients in this study were followed up monthly by clinical examination and every 3 months by sonomammography to diseased and healthy breasts, as well as by chest X-ray and abdominopelvic ultrasound. At the end of followup, findings of Her2/neo expression, p53, and hormonal receptor status, were correlated for patients' response to neoadjuvant chemotherapy, and for 3-year DFS and OAS rates. Relation of Her2/neu over-expression to p53, hormonal receptor status and conventional prognostic factors was analyzed.

## 3. Results

The 95 cases included in the present study showed an age incidence which ranged from 33 to 72 years, with the median age of 48 years. In fifty-seven cases, 60% were of <50 years and the other 38 cases (40%) were ≥50 years of age. The majority of patients had grade II tumors (65 patients; 68.4%) and had large tumor size (>5 cm) (86 patients, (90.5%), 19 patients of them had no palpable ipsilateral axillary nodes (N0), 55 patients had mobile ipsilateral axillary nodes (N1 disease), and only 21 patients had fixed nodes (N2 disease)). Seventy four patients had both ER and PR positive breast cancer. HER-2/neu positive staining was found in 20 (21.1%) patients, and p53 inactivation in 29 (31.5%) patients (Table 1). 

Out of the 95 patients, 68 patients (71.6%) showed partial response and underwent CBS with negative margins and the remaining 27 patients underwent MRM. The association of clinical response and the findings of hormonal receptor, HER-2, and p53 protein were evaluated. The hormonal receptors were positive in 77.6% of patients with OR and in 78.9% of NR patients (*P* = 1; Fisher's exact test). There were no HER-2 and p53, protein expression significant differences before chemotherapy between the OR and NR groups (*P* = 0.345 and *P* = 0.096, resp., Fisher exact test) (Table 2).

In terms of relapse following conservative surgery with negative margins (68 patients), 12 cases had a disease relapse (17.6), 4 of them had isolated local recurrence and were salvaged by mastectomy. Following MRM (27 patients), there were 7 disease relapses 3 of them were isolated relapse and were surgically resected (Table 3). 

**Figure 1:**
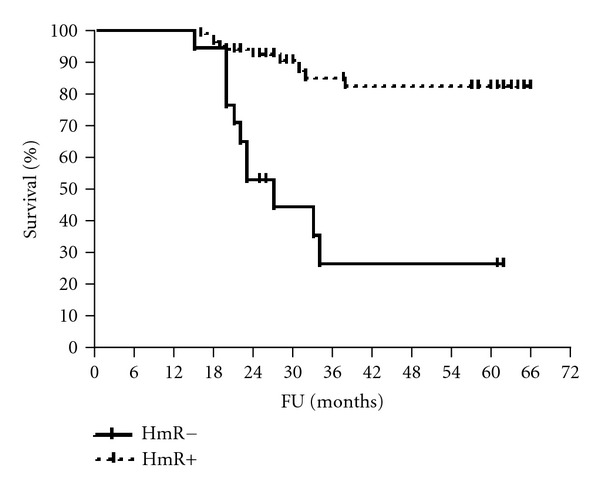
DFS of patients according to HmR status.

**Figure 2:**
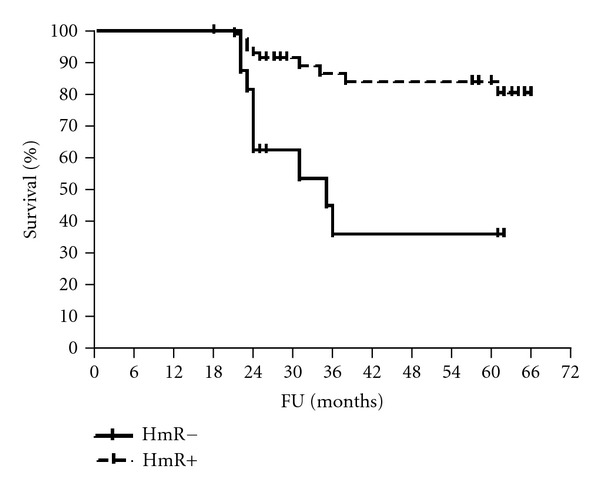
OAS of patients according to HmR status.

**Figure 3:**
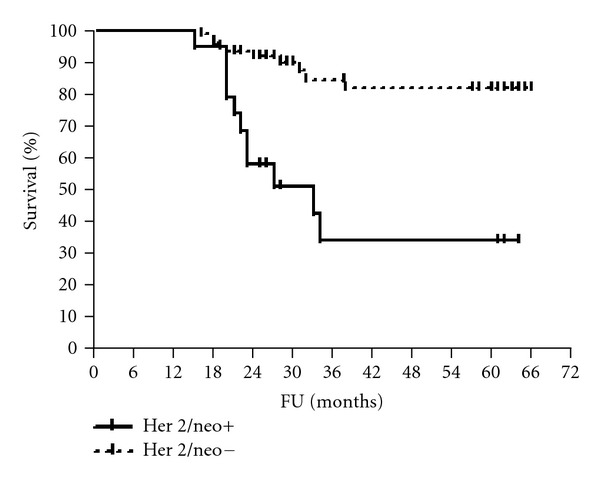
DFS of patients according to Her2/neu status.

**Figure 4:**
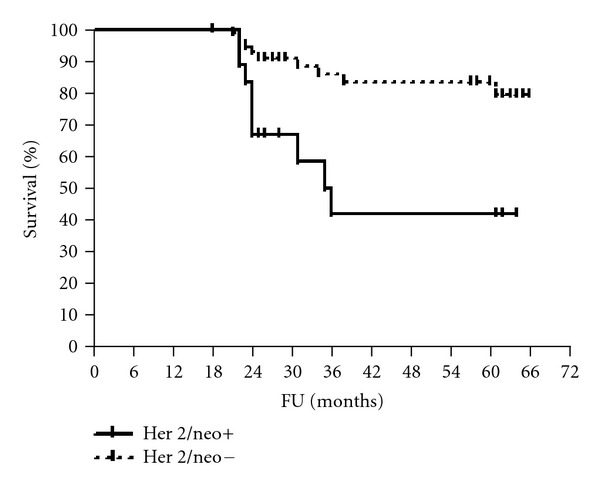
OAS of patients according to Her2/neu status.

**Figure 5:**
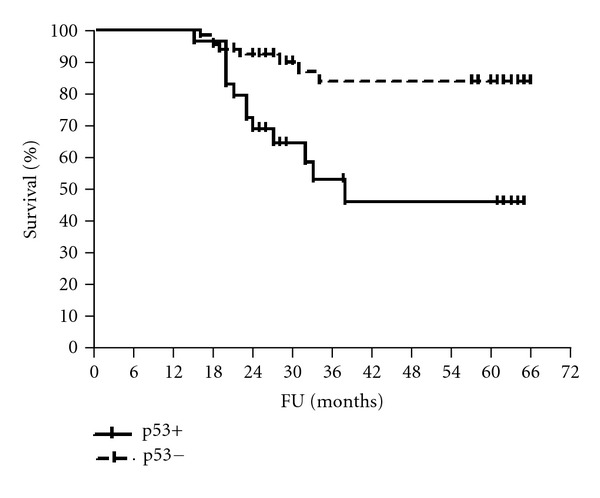
DFS of patients according to p53 status.

**Figure 6:**
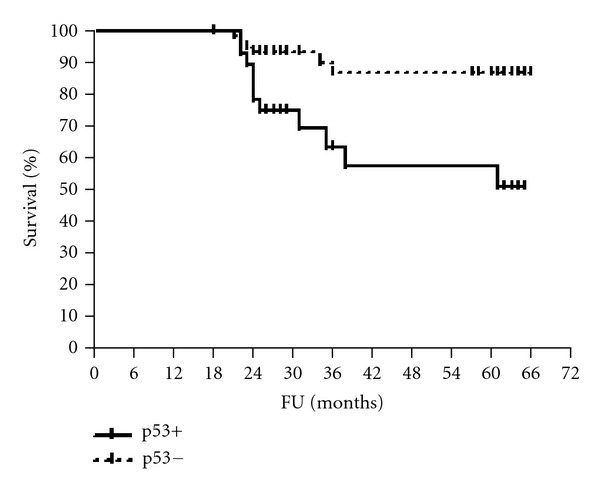
OAS of patients according to p53 status.

The median follow-up period was 61 months (range 56–65). The 5-year disease-free and overall survival rates were calculated for patients according to HmR status, Her2/neu expression, and p53 staining (Figures 1, 2, 3, 4, 5, and 6). The DFS rates at 5 years were 82.3% and 26.5% for breast cancer patients with positive and negative hormonal receptors, respectively (*P* < 0.0001, HR 21.48, 95% C.I.: 6.34–72.72), whereas the OAS rates were 84% and 35.7%, respectively (*P* = 0.0001, HR 11.59, 95% C.I.: 3.32–40.47). The 5-year DFS rates were 33.8% and 81.8% for patients with Her2+ and Her2− breast cancer, respectively (*P* < 0.0001, HR 12.27, 95% C.I.: 3.92–38.42), and the 5-year OAS rates were 41.7% and 83.2%, respectively (*P* = 0.001, HR 7.14, 95% C.I.: 2.21–23.06). For patients with inactive p53 and those with intact p53 breast cancer, the DFS rates were 46.1% and 83.6% respectively (*P* = 0.0007, HR 5.15, 95% C.I.: 1.99–13.33), and OAS rates were 57.1% and 86.7% respectively (*P* = 0.0029, HR 4.81, 95% C.I.: 1.71–13.52) (Table 4).

Hormonal receptors were positive in 74 cases (77.9%), where 72 cases (97.3%) of these were HER-2 negative. On the other hand, 21 cases (22.1%) were HR negative, 18 of them (85.7%) were positive for Her-2 over-expression. Her-2 over-expression was statistically significant associated with loss of HmR positive immuno-staining (*P* < 0.0001), grade III breast cancer (*P* < 0.0001), advanced lymph node status (*P* = 0.0039), and patient <50 years of age (*P* = 0.0108) (Table 5). On the other hand, Her-2 over-expression was not statistically significant associated with p53 inactivation (*P* = 0.412) and large tumor size (≥5 cm) (*P* = 1). 

 With respect to biological features, all patients (*n* = 9) with inactive p53 and Her-2/neu coexpressing tumors and 8 out of 11 breast cancer patients (72.7%) with Her2/neu expression and intact p53 were negative for hormonal receptors (Table 6). 

## 4. Discussion

Identifying pretreatment factors that predict chemotherapy sensitivity would potentially allow for individually tailored chemotherapy. Using prognostic factor assessment to guide the selection of therapy has been explored with both adjuvant and neoadjuvant therapy regimens. Results have been discordant due to the heterogeneity of chemotherapy evaluated. Both taxanes and anthracycline have currently been used in neoadjuvant breast cancer treatment [17]. Investigations focusing on conventional markers did not allow to make valid predictions and were not able to find correlations between classical markers and response to taxanes and anthracyclines [18, 19]. A wide variety of morphology-based and molecular-based breast cancer prognostic factors have been studied. An expanded understanding of the biology of breast cancer has led to the identification of the Her-2/neu receptor [11, 20] and p53 [21]. In the present study, the incidences of unfavorable biological tumor characteristics as negative hormonal receptors, Her2/neu overexpression, and inactive p53 were 18%, 20%, and 31% respectively. These figures are comparable with those in the reported studies. Li et al. [22] have documented a prevalence of 21% of hormonal receptor negative breast cancer patients in US from 1992 to 1998. Her2/neu overexpression has been identified in 10% to 34% of patients with breast cancer [7, 23]. Mutations in the tumor suppressor gene p53 are present in 18%–25% of primary breast carcinomas [24].

The role of hormone receptor status as a predictive marker is less clear. In our study, negative hormonal receptors, expression of the Her-2 protein, and positive p53 staining were not able to predict the clinical tumor response. When it was evaluated in some trials of neoadjuvant chemotherapy, including docetaxel alone and doxorubicin followed by docetaxel, hormonal receptor status was not associated with clinical response [25]. Conversely, two clinical studies reported that negative hormonal receptor status was correlated with chemosensitivity [26, 27]. The role of Her-2 as a predictive factor was studied [28, 29], where a correlation between Her-2 expression and response to neoadjuvant chemotherapy could not be observed in patients with breast carcinoma treated with 5-fluorouracil, doxorubicin, and cyclophosphamide combination (FAC regimen). In a meta-analysis of immunohistochemically evaluated p53 expression of more than 9000 breast cancer patients [30], the prognostic and predictive value of p53 overexpression appeared weak [31]. 

In the present study, the rate of isolated local recurrence was 7.3% (7 out of 95 patients). This is confirmed by a recent retrospective study conducted by Shen et al., [32] who investigated patients with T4 locally advanced breast cancer treated with 4 cycles of NAC. The actuarial breast cancer recurrence rate was rather low with 6%.

The present study showed that the 5-year DFS and OAS rates of patients with hormonal receptor positive breast cancer were statistically significant and were higher than those in patients with negative hormonal receptors. These findings are confirmed by recent studies which have shown survival advantages among women with hormone receptors positive tumors relative to women with hormone receptor negative tumors [33–37]. 

Her2/neu overexpression is considered as a major prognostic factor in stage II and III breast cancer patients treated with neoadjuvant taxane, and anthracycline combination chemotherapy [9, 23, 38–41]. In the current study, there were statistically significant disease-free and overall survival advantages in patients with Her2 negative breast cancer in comparison with those in Her2 positive breast cancer patients. These results are reinforced by results of an Indian study [38]. Tiezzi et al. [39] reported that patients with Her-2 positive tumors achieved an overall survival rate of 25% after 48 months in contrast with 70% in patients with Her-2 negative expression. The 5-year DFS and OAS rates were significantly higher in p53 intact breast cancer patients than that in p53 inactive cancer patients, in the present study. This is in agreement with Gimenez et al. [42] but is in contrast to recent studies [24, 25], where overall survival and relapse-free survival rates were not independently predictive by p53 status in the overall patients group. 

In the present study, Her2 overexpression was an associated feature of negative hormonal receptors, higher tumor grade, higher number of invaded axillary lymph nodes, and younger age at diagnosis. This is confirmed by many studies [40, 41, 43, 44]. Nieto et al. [43] demonstrated that alteration of Her2/neu is an associated feature of tumor aggressiveness as absence of hormonal receptors, advanced tumor stage, and young age at diagnosis. An inverse association had been found between Her-2/neu over-expression and the presence of receptors for steroid hormones (estrogen and progesterone) in both clinical correlative studies and experimental models [40]. Her-2/neu overexpression was significantly associated with tumor grade [41] and increasing number of involved axillary lymph nodes [44]. 

In contrast with the Rashed et al. [40], the present study did not show significant association between Her2 overexpression and tumor size. This could be explained by the higher proportion of large tumor size (>5 cm) in our study (86 out of 95 patients; 90.5%) than that in the reported study (18 out of 50 patients; 36%). 

In the current study, inactive p53 and Her2/neu coexpressing tumors represented 9.5% (9 out of 95 patients) and were more often negative for hormonal receptors. This is comparable with the rate of breast cancer with coexpression reported in previous studies which ranged between 3% and 19.5% of total breast cancers examined [10, 45] and has been reported to be prognostically unfavorable [46].

*In Conclusion* we have found that the studied biological factors were not able to predict tumor response to neoadjuvant chemotherapy. However, Her2/neu overexpression was associated with poor disease-free and overall survival rates, which may be explained on the ground that it was significantly associated with negative hormonal receptors and high tumor grade.

## Figures and Tables

**Table 1:** Patients' characteristics.

Variable	Baseline characteristics of all patients (*n* = 95)	
	Number	%
Age at diagnosis		
<50 years	57	60
≥50 years	38	40
Clinical tumor size		
<5 cm	9	9.5
≥5 cm	86	90.5
Lymph node status		
N0	19	20
N1	55	55.6
N2	21	22.1
Histologic grade		
grade II	65	68.4
grade III	30	31.6
HmR Status		
HmR positive	74	77.9
HmR negative	21	22.1
Her2/neu status		
Her2/neu+	20	21.1
Her2/neu−	75	78.9
P53 status		
Inactive p53	29	31.5
Intact p53	66	69.5

Total	95	100

**Table 2:** Response of patients of locally advanced breast cancer to neoadjuvant chemotherapy according to biological prognostic factors.

Variable	Objective response (*n* = 76)		No response (*n* = 19)		*P* value
	Number	%	Number	%
HmR status					1
HmR+ (*n* = 74)	59	77.6	15	78.9	
HmR− (*n* = 21)	17	22.3	4	21.1	
Her2/neo status					0.345
Her2/neo+ (*n* = 20)	18	23.7	2	10.5	
Her2/neo− (*n* = 75)	58	76.3	17	89.5	
p53 status					0.096
Inactive p53 (*n* = 29)	20	26.3	9	47.4	
Intact p53 (*n* = 66)	56	73.7	10	52.6	

**Table 3:** Pattern of disease relapse in all patients.

Disease relapse	CBS (*n* = 68)		MRM (*n* = 27)		Total (*n* = 95)	
	Number	%	Number	%	Number	%
LR	4	5.9	3	11.1	7	7.3
LR + DM	1	1.5	2	7.4	3	3.2
DM	7	10.3	2	7.4	9	9.5

Total	12	17.7	7	25.9	19	20

**Table 4:** Five-year OAS and DFS rates in 95 breast cancer patients according to biological prognostic factors.

Variable	5-year DFS rate (%)	5-year OAS rate (%)
Hormonal receptor (HmR) status		
HmR+ (*n* = 74)	82.3	84
HmR− (*n* = 21)	26.5	35.7

*P* value	*P* < 0.0001 HR 21.48 95% CI 6.34–72.72	*P* = 0.0001 HR 11.59, 95% CI 3.32–40.47

Her2/neo status		
Her2/neo+ (*n* = 20)	33.8	41.7
Her2/neo− (*n* = 75)	81.8	83.2

*P* value	*P* < 0.0001 HR 12.27, 95% CI 3.92–38.42	*P* = 0.001 HR 7.14, 95% CI 2.21–23.06

p53 status		
Inactive p53 (*n* = 29)	46.1	57.1
Intact p53 (*n* = 66)	83.6	86.7

*P* value	*P* = 0.0007 HR 5.15, 95% CI 1.99–13.33	*P* = 0.0029 HR 4.81, 95% CI 1.71–13.52

**Table 5:** Relation of Her 2/neu to p53, hormonal receptor status, grade, tumor size, lymph node status, and patients' age.

Variable	Number	Her2/neu+ (*n* = 20)		Her2/neu− (*n* = 75)		*P* value
		Number	%	Number	%
Hormonal receptor (HmR)						<0.0001
HmR+	74	2	2.7	72	97.3	
HmR−	21	18	85.7	3	14.3	
p53 status						=0.412
Inactive p53	29	8	27.6	21	72.4	
Intact P53	66	12	18.2	54	81.8	
Grade						<0.0001
G II	65	4	6.2	61	93.8	
G III	30	16	53.3	14	47.7	
Tumor size						=1
<5 cm	9	2	22.2	7	77.8	
≥5 cm	86	18	20.1	68	79.9	
Lymph node status						=0.0039
N0	19	0	0	19	100	
N1	55	11	20	44	80	
N2	21	9	42.9	12	57.1	
Age						=0.0108
<50 years	57	17	29.8	40	70.2	
≥50 years	38	3	7.9	35	92.1	

**Table 6:** Coexpression of Her2/neu and p53 according to HmR status.

variable	Her2/neu+ and inactive p53	Her2/neu− and intact p53	Her2/neu+ and intact p53	Her2/neu− and inactive p53	Total	*P* value
HmR+	0	51	3	20	74	<0.0001
HmR−	9	3	8	1	21	

Total	9	54	11	21	95	

## Abbreviations

CBS: Conservative breast surgery
MRM: Modified radical mastectomy
Gys: Grays
Mev: Million electron volt
OAS: Over all survival
DFS: Disease free survival
NAC: Neoadjuvant chemotherapy
LABC: Locally advanced breast cancer
ER: Estrogen receptor
PR: Progesterone receptor
HmR: Hormonal receptor
HR: Hazard ratio
CI: Confidence interval
2D: Two dimensional
3 D: Three dimensional
LVEF: Left ventricular ejection fraction
ELISA: Enzyme linked immunosobent assay
*μ*L: Microlitre
